# Canine Mesenchymal Stromal Cell-Mediated Bone Regeneration is Enhanced in the Presence of Sub-Therapeutic Concentrations of BMP-2 in a Murine Calvarial Defect Model

**DOI:** 10.3389/fbioe.2021.764703

**Published:** 2021-11-02

**Authors:** Lauren K. Dobson, Suzanne Zeitouni, Eoin P. McNeill, Robert N. Bearden, Carl A. Gregory, W. Brian Saunders

**Affiliations:** ^1^ Department of Small Animal Clinical Sciences, College of Veterinary Medicine and Biomedical Sciences, Texas A&M University, College Station, TX, United States; ^2^ Department of Molecular and Cellular Medicine, Institute for Regenerative Medicine, Texas A&M Health Science Center, College Station, TX, United States

**Keywords:** canine, bone, regeneration, calvarial, BMP-2, *in vivo*, MSC

## Abstract

Novel bone regeneration strategies often show promise in rodent models yet are unable to successfully translate to clinical therapy. Sheep, goats, and dogs are used as translational models in preparation for human clinical trials. While human MSCs (hMSCs) undergo osteogenesis in response to well-defined protocols, canine MSCs (cMSCs) are more incompletely characterized. Prior work suggests that cMSCs require additional agonists such as IGF-1, NELL-1, or BMP-2 to undergo robust osteogenic differentiation *in vitro*. When compared directly to hMSCs, cMSCs perform poorly *in vivo*. Thus, from both mechanistic and clinical perspectives, cMSC and hMSC-mediated bone regeneration may differ. The objectives of this study were twofold. The first was to determine if previous *in vitro* findings regarding cMSC osteogenesis were substantiated *in vivo* using an established murine calvarial defect model. The second was to assess *in vitro* ALP activity and endogenous BMP-2 gene expression in both canine and human MSCs. Calvarial defects (4 mm) were treated with cMSCs, sub-therapeutic BMP-2, or the combination of cMSCs and sub-therapeutic BMP-2. At 28 days, while there was increased healing in defects treated with cMSCs, defects treated with cMSCs and BMP-2 exhibited the greatest degree of bone healing as determined by quantitative μCT and histology. Using species-specific qPCR, cMSCs were not detected in relevant numbers 10 days after implantation, suggesting that bone healing was mediated by anabolic cMSC or ECM-driven cues and not *via* engraftment of cMSCs. In support of this finding, defects treated with cMSC + BMP-2 exhibited robust deposition of Collagens I, III, and VI using immunofluorescence. Importantly, cMSCs exhibited minimal ALP activity unless cultured in the presence of BMP-2 and did not express endogenous canine BMP-2 under any condition. In contrast, human MSCs exhibited robust ALP activity in all conditions and expressed human BMP-2 when cultured in control and osteoinduction media. This is the first *in vivo* study in support of previous *in vitro* findings regarding cMSC osteogenesis, namely that cMSCs require additional agonists to initiate robust osteogenesis. These findings are highly relevant to translational cell-based bone healing studies and represent an important finding for the field of canine MSC-mediated bone regeneration.

## Introduction

Non-union bone defects are a major challenge in orthopaedics. It has previously been estimated that approximately 10% of fractures result in non-union (Marsh 1998). In some patients, poor bone quality and inadequate fixation provide insufficient biomechanical stabilization and result in non-union (Bishop et al., 2012). In other patients, age and other co-morbidities lead to impaired endogenous bone repair and an insufficient biologic response to achieve union (Hak et al., 2014). In many patients, a combination of biomechanical and biologic factors contribute equally to the development of non-unions. Development of methods to achieve rapid, reliable healing of large bone defects will reduce the incidence of implant failure, reoperation, patient-morbidity, and the burden on the healthcare system.

For decades, autografts have been the gold standard for treatment and prevention of non-unions due to their ability to provide osteogenic, osteoinductive, and osteoconductive stimuli to the defect (Kao and Scott 2007). Disadvantages of autografting include a paucity of osteogenic cells, limited osteoconductivity, poor host-cell adhesion properties, insufficient biomechanical properties, incomplete osteointegration, and untoward immune response (Boden, 2002; Cuomo et al., 2009). Therefore, a clear need exists for the development of an effective technology with efficacy similar to autografting but without the associated limitations. The most feasible form for this technology is a cell-scaffold composite; however, achieving clinical efficacy, reproducibility, biocompatibility, and an effective production pipeline have hampered development of this strategy (McNeill et al., 2020).

Mesenchymal stromal cells (MSCs) are promising agents for bone repair and regeneration. MSCs isolated from adult tissues such as bone marrow, synovium, adipose, and other tissues possess the capacity to differentiate into numerous connective tissue lineages including osteoblasts, adipocytes, and chondrocytes. MSCs also possess the ability to provide stromal support through deposition of anabolic matrices, deliver trophic factors to repairing tissues, and exhibit immunomodulatory properties. However, the use of human MSCs for bone repair in animal models and the clinical setting has been disappointing due to donor variability and inconsistent outcomes. For these reasons, much work is in progress to optimize the osteoregenerative potential of MSCs via modulation of the canonical Wnt pathway, co-administration of MSCs with osteogenic extracellular matrices, and the use of iPS-derived MSCs (Zeitouni et al., 2012; McNeill et al., 2020).

A major hurdle for novel MSC-scaffold constructs is the successful translation from *in vitro* and rodent model findings to demonstration of efficacy in clinical patients. Large animal models are often utilized as an intermediary toward clinical therapy in human beings (Hatsushika et al., 2014; Cong et al., 2019). While sheep, pig, dog, and goat models are well-described, the dog represents an under-represented model species for orthopaedic regenerative studies (Hoffman and Dow 2016). Dogs exhibit similar orthopaedic physiology and biomechanics to human beings, have complex immune systems, are recognized by regulatory agencies, exhibit more genetic diversity than domestic livestock species, and are amenable to functional outcome measures such as gait analysis. Another advantage of the dog as a translational model species is the ability to perform veterinary clinical trials using naturally occurring non-union fractures with outcome measures identical to future human clinical trials. Moreover, there is precedent for using naturally occurring diseases in the canine model to evaluate novel therapeutics for use in human beings (Hubbard et al., 2018).

While hMSCs have been intensely characterized and consistently undergo *in vitro* osteogenesis in response to well-defined differentiation protocols, a relative handful of studies have focused on canine MSC [cMSCs; (Volk et al., 2005; Levi et al., 2011; Bearden et al., 2017; James et al., 2017; Gasson et al., 2021)]. In regards to osteogenic differentiation, a growing body of work suggests that cMSCs do not respond to hMSC osteogenic protocols without addition of agonists such as bone morphogenic protein 2 (BMP-2), insulin-like growth factor-1 (IGF-1), or neural epidermal growth factor-like 1 protein [NELL-1; (Volk et al., 2005; Levi et al., 2011; Bearden et al., 2017; James et al., 2017; Gasson et al., 2021)]. Importantly, the reduced *in vitro* performance of cMSCs was previously documented *in vivo* in a nude mouse calvarial defect model (Levi et al., 2011). Defects treated with human adipose-derived MSCs exhibited significant bone healing, whereas defects treated with murine or canine MSCs exhibited minimal bone healing and were not different than untreated controls (Levi et al., 2011). These results suggest that the mechanisms that control osteogenesis in cMSCs and hMSCs may be somewhat different.

BMP-2 has undoubtedly demonstrated the most clinical promise as an alternative to autografting (Boden et al., 2000; Hecht et al., 2000; Liu et al., 2019). When applied on a compression resistant calcium phosphate matrix, recombinant human BMP-2 (rhBMP-2) enhances lumbar spinal fusions and improves long-bone fracture healing (Glassman et al., 2005; Dimar et al., 2006). Importantly, rhBMP-2 is also effective in other species such as sheep and dogs has been used in canine bone healing models (Kandziora et al., 2002; Schmoekel et al., 2004; Kinsella et al., 2011). Concerns have surfaced with BMP-2 therapy due to reports of uncontrollable/ectopic bone formation, respiratory difficulty, osteolysis, cervical and soft tissue inflammation, adipogenic activation, urogenital events and bone cyst formation (Carragee, Hurwitz, and Weiner 2011; Faundez et al., 2016). As such, efforts are underway to refine BMP-2 dosing or develop alternative BMP-2 treatment strategies. Using a rat lumbar spinal fusion model, Bae and colleagues demonstrated that delivery of a sub-therapeutic concentration of BMP-2 in combination with fresh bone marrow allografts led to improved lumbar fusion rates, suggesting that cells could be combined with a low dose of BMP-2 to achieve clinical unions (Bae et al., 2013).

Given the prior studies demonstrating that cMSCs fail to undergo robust *in vitro* osteogenesis without supplemental BMP-2 *in vitro* (Volk et al., 2005; Levi et al., 2011; Bearden et al., 2017; James et al., 2017; Gasson et al., 2021), the objective of this study was to determine whether bone marrow-derived cMSCs were capable of inducing a healing response in an established murine calvarial defect model in the absence or presence of BMP-2, and to determine whether a combination of sub-therapeutic rhBMP-2 and cMSCs behaved synergistically similar to the findings of Bae et al. We hypothesized that cMSCs co-administered with a sub-therapeutic concentration of rhBMP-2 would induce superior bone healing as compared to untreated defects or defects treated with either cMSCs or sub-therapeutic rhBMP-2 alone.

## Materials and Methods

### Culture and Differentiation of cMSCs and hMSCs

**FIGURE 1:**
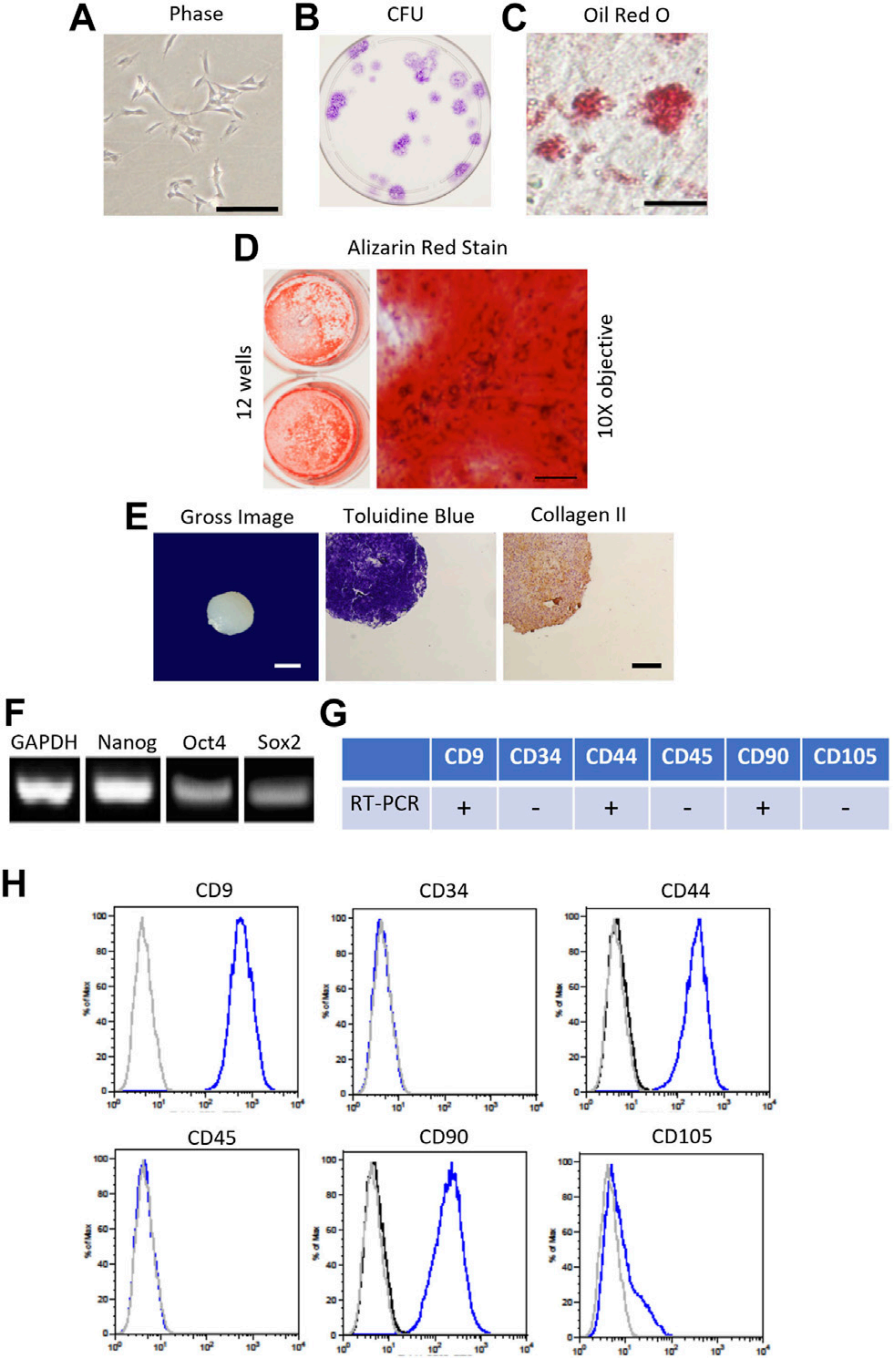
Characterization of canine bone marrow-derived mesenchymal stromal cell (cMSC). **(A)**: Passage 1 cMSCs were expanded at 100 cells/cm^2^ in CCM with media exchange every 48 h until cMSCs reached 70–80% Representative phase contrast microscopy image of cMSCs exhibiting a spindle-shaped, mesenchymal phenotype (bar = 250 µm). **(B)**: cMSCs were assessed with the classic colony forming unit (CFU) assay to demonstrate ability to form distinct colonies in the absence of media exchange over a 21 days time course. **(C)**: Adipogenic differentiation was confirmed *via* Oil Red O staining after 21 days of culture in adipogenic differentiation medium (bar = 25 µm). **(D)**: Osteogenic differentiation was confirmed *via* Alizarin Red Stain after 21 days of culture in osteogenic differentiation medium (ODM) supplemented with + 200 ng/ml of rh-BMP-2. Gross photography of representative wells are provided (left) in conjunction with transmitted light microscopy images (right, bar = 125 µm). **(E)**: Chondrogenic differentiation was confirmed using the traditional micromass assay. Gross image of a representative cMSC chondrogenic spheroid (bar = 300 µm) is provided on the left, with Toludine Blue for and Collagen Type II immunohistochemistry shown at center and right (bar = 150 µm). **(F)**: cMSCs were assessed for endogenous expression of the plasticity-associated genes Nanog, Oct4, and Sox2 using RT-PCR. Canine GAPDH was used as a housekeeping gene. **(G, H)**: Real-time PCR and flow cytometry analysis of cMSCs demonstrating expression of CD9, CD44, and CD90 and the absence of CD34, CD45, and CD105. Using these characterization methods, the cMSCs met the criteria for MSCs.

Canine MSCs were obtained from a bone marrow aspirate from the proximal humerus of a haematologically healthy canine donor under an approved Animal Use Protocol (2011-149) with the supervision of the Institutional Animal Care and Use Committee at Texas A&M University. Using Ficoll centrifugation and plastic adherence, mononuclear cells were plated at 30,000 cells/cm^2^ on 150 cm^2^ dishes to isolate Passage 0 (P0) cells in complete culture medium (CCM) consisting of alpha minimal essential medium (α-MEM, Invitrogen, Waltham, MA) containing 10% premium select fetal bovine serum (Atlanta Biologicals, Norcross, GA), 2 mM L-glutamine, 100 units/ml penicillin, and 100 ug/ml streptomycin (Invitrogen) (Bearden et al., 2017). Plates were washed daily with PBS for 3 days and media exchange was performed at 48 h intervals until colonies of P0 cells were visible. Cells were washed, trypsinized, and Passage 1 (P1) cells were expanded at clonal density 100 cells/cm^2^ in CCM with media exchange every 48 h until cells reached 70% confluence. Cells were characterized and confirmed to be cMSCs as defined by Dominici with slight modifications for the canine species Figure 1; (Dominici et al., 2006; Bearden et al., 2017). The preparation of cMSCs used in the present study was selected due to its representative performance in osteogenic differentiation assays (Bearden et al., 2017). P1 cells were cryopreserved in 1 × 10^6^/ml aliquots in α-ΜΕΜ with 30% FBS and 5% DMSO (Hybrimax, Sigma-Aldrich, St. Louis, MO). For subsequent experiments, cells were thawed and expanded to Passage 2 (P2) cells at 100 cells/cm^2^ in CCM.

Human MSCs (hMSCs) were obtained from the iliac crest of two haematologically healthy human donors under a Texas A&M/Baylor Scott and White Hospital (Temple, TX) Institutional Review Board-approved protocol. hMSCs were isolated, expanded, and cryopreserved using identical methods to the cMSCs, with the exception that the CCM used to isolate and expand the hMSCs contained 20% premium select FBS (Atlanta Biologicals). The hMSCs were selected for the present study due to their representative performance in osteogenic differentiation assays (Clough et al., 2015).

### Murine Calvarial Defect Model

Murine calvarial defect studies were conducted under an approved AUP (2016-0144) with the supervision of the Texas A&M University Institutional Animal Care and Use Committee. 60-days female Nu/J mice were acquired from Jackson Laboratories (Bar Harbor, ME) and housed in sterile conditions. Mice (*n* = 10/group) were anesthetized using isoflurane inhalant anesthesia in 100% O_2_ with whole body warming at 37°C. Post-operative care consisted of buprenex administration twice a day for 3 days and a hydrogel diet to encourage caloric intake. After sterile surgical preparation with chlorhexidine and alcohol, 4 mm unilateral calvarial defects were created with an osteotomy burr (Roboz Surgical, Gaithersburg, MD) under continuous saline irrigation. To ensure consistent defect placement, each defect was created 1–2 mm from the sagittal and coronal sutures.

**FIGURE 2:**
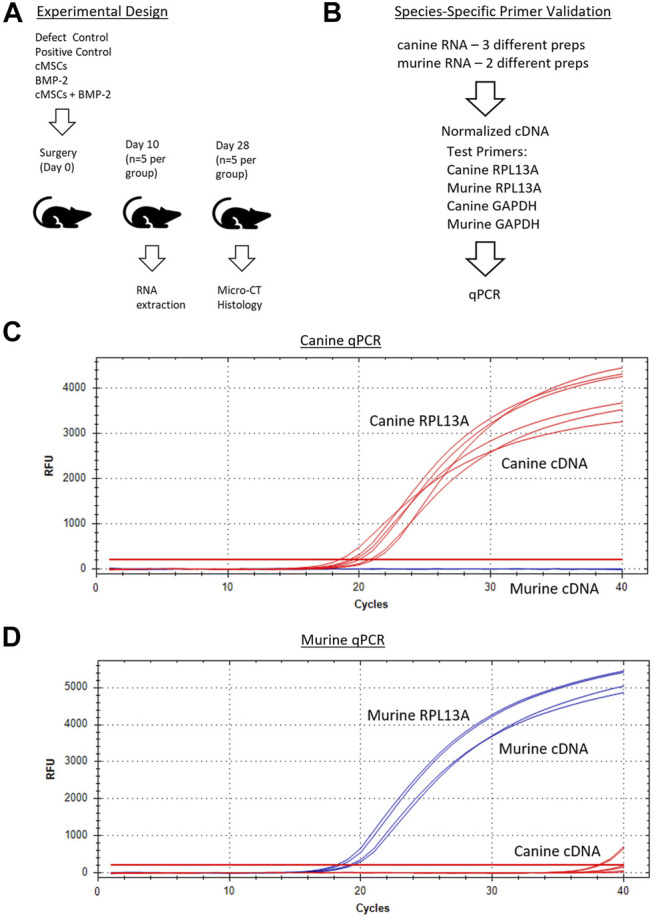
Overall experimental design and species-specific primer development and validation. **(A)**: Surgical treatment groups included a negative control (defect control), positive control (100 μg/ml BMP-2), 2 × 10^6^ cMSCs, sub-therapeutic BMP-2 (6 μg/ml), or cMSCs + BMP-2 (6 μg/ml). Mice were terminated for RNA extraction (Day 10) or μCT and histology (Day 28). **(B)**: qPCR primer design and validation schematic for canine and murine RNA samples. Primer pairs that amplified RPL13A and GAPDH in a species-dependent manner were selected for further use. **(C, D)**: Canine and murine RPL13a qPCR amplification curves for canine cDNA (red lines) and murine cDNA (blue lines). Canine and murine RPL13a primer-pairs resulted in species-specific amplification and were used in subsequent analyses.

P1 cMSCs were thawed, washed, plated at 100 cells/cm^2^, and cultured in CCM until P2 cells were 70–80% confluent on the day of surgical implantation. P2 cMSCs were washed in PBS, trypsinized, neutralized with CCM, washed in α-MEM to remove residual serum, and suspended in sterile filtered PBS in 2 × 10^6^ cell aliquots per defect. cMSC aliquots were stored on ice in the surgical suite until the time of administration. Calvarial defects were treated with either 2 × 10^6^ cMSCs in murine plasma, 6 μg/ml recombinant human BMP-2 (rhBMP-2, R and D systems, Minneapolis, MN) in murine plasma [BMP-2; (Bae et al., 2013)], or the combination of 2 × 10^6^ cMSCs and 6 μg/ml rhBMP-2 in murine plasma (cMSCs + BMP-2; Figure 2A). At the time of defect creation, the aliquots of cMSCs were centrifuged, resuspended in 20 µL of 4°C murine plasma (Sigma-Aldrich), added to 20 µL of 4°C 2X thromboplastin C (Fisher Scientific, Waltham, MA), and immediately pipetted onto each calvarial defect. For the cMSC treatment groups that also received 6 µg/ml rhBMP-2, after combination of murine plasma and thromboplastin, 6 µL of rhBMP-2 was added to the pipette for implantation into the defect. Gelation of the murine plasma was confirmed by manual agitation of the solution with the pipette tip. A positive control group received 100 μg/ml rhBMP-2 on a gelatin surgical sponge (positive control) based on previous work (Bae et al., 2013) (Gelfoam, Baxter International, Deerfield, IL). A negative control group [defect control group (*n* = 4 mice/group)] did not receive inductive treatment but did receive 40 µL of plasma and thromboplastin to confirm that co-administration of murine plasma had no effect on defect healing. Mice were recovered from anesthesia and housed in group cages until termination at Day 10 (RNA isolation and quantification of cMSCs) or Day 28 (assessment of healing *via* microCT and histology; Figure 2A).

### Canine and Murine Cell Titration Standard Curve

In order to screen calvarial defects for the presence of canine cells post-implantation, murine and canine RNA were first used to create species-specific primers for real-time quantitative PCR (qPCR). Murine fibroblasts (C3H10T1/2, ATCC, Manassas, VA) and cMSCs were cultured in CCM. Two preparations of C3H10T1/2 and three preparations of cMSCs (1 × 10^6^ cells/prep) were isolated and used to extract RNA using the High Pure RNA Isolation Kit (Roche Diagnostics, Basel, Switzerland). RNA samples were quantified *via* nanodrop (Thermofisher, Waltham, MA) to determine RNA yields and were normalized to identical RNA values prior to generation of cDNA (SuperScript III cDNA kit, Invitrogen). For qPCR, cDNA was amplified in a 20 µL reaction containing SYBR Green PCR master mix (Fast SYBR Green, Applied Biosystems, City, State) on a CFX96 Real-Time System (Bio-Rad, City, State). Canine RPL13A and GAPDH (Peters et al., 2007), murine RPL13A and GAPDH made using primer-blast (Ye et al., 2012), were used to create species-specific housekeeping primers (Sigma, St. Louis, MO; Figure 2B). The two murine and three canine cDNA preparations were assessed in duplicate with qPCR using both murine and canine RPL13A and GAPDH (4 primer pairs) to confirm species specificity.

**FIGURE 3:**
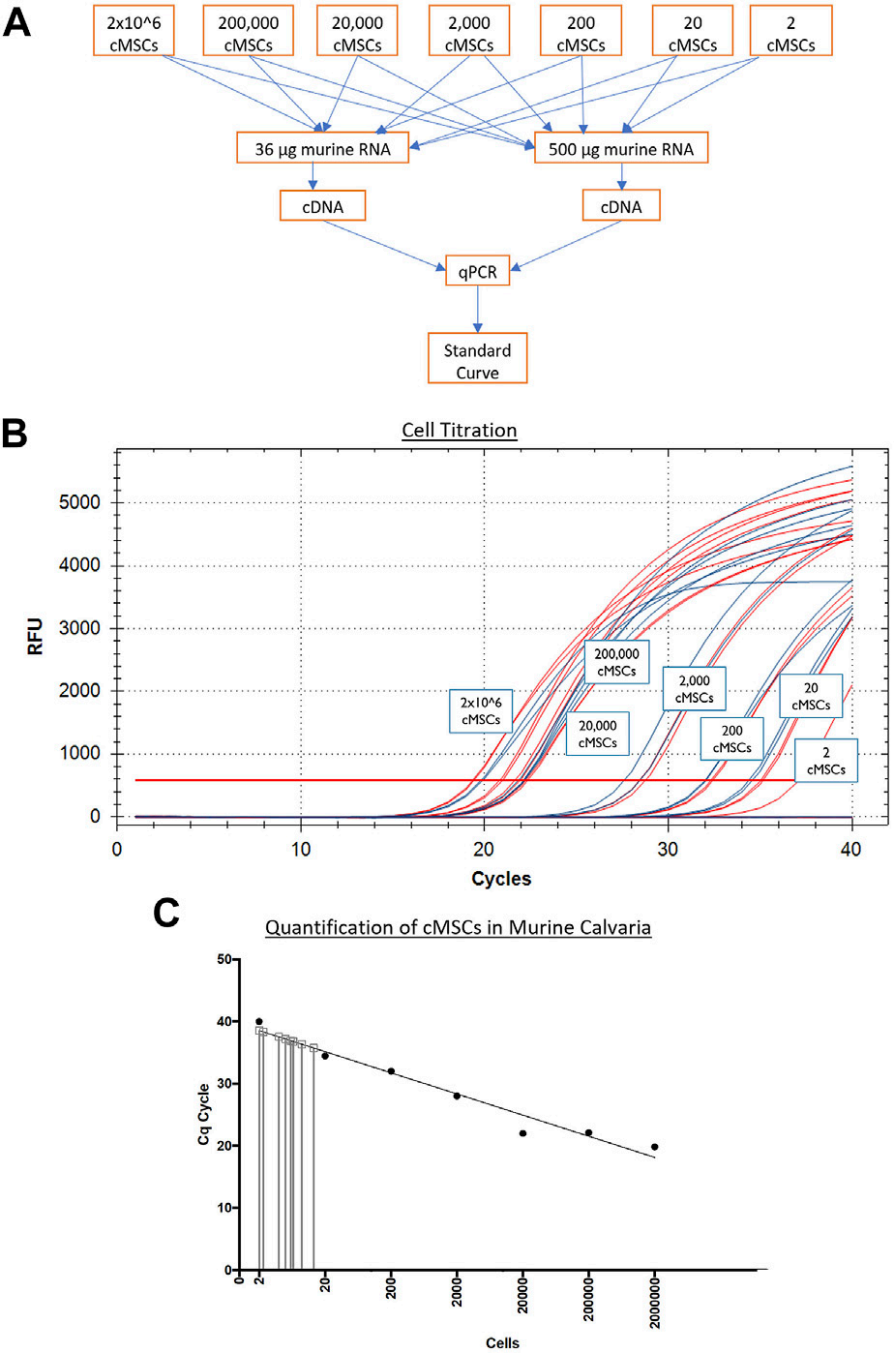
Generation of a qPCR-based cMSC standard curve to detect cMSCs in calvarial defects at Day 10. **(A)**: Serial dilutions of RNA were performed on RNA extracted from 2 × 10^6^ total cMSCs. RNA samples were added to individual sample tubes containing either 36 or 500 μg of murine RNA to determine if the amount of murine RNA would affect the ability to identify canine RPL13A. cDNA samples were generated and qPCR was performed. **(B)**: qPCR amplification curves are shown for canine RPL13A from cDNA samples generated with either 36 μg (red lines) or 500 μg (blue lines) of murine RNA. **(C)**: Cycle thresholds from Panel A (red lines) were used to generate a standard curve using a linear curve fit technique. RNA samples were isolated from the calvarial defects treated with cMSCs or cMSCs + BMP-2. These samples contained both murine and canine RNA. cDNA samples were generated and qPCR performed for canine RPL13A. Cycle threshold results were interpolated on the standard curve. At Day 10 post implantation, only 2–20 total cMSCs were detected.

Next, murine and canine RNA were used to determine a correlation of threshold cycles and create a standard curve for minimum and maximum numbers of detectable cells. Murine fibroblasts (C3H10T1/2) or cMSCs (2 × 10^6^) were used to extract RNA using the High Pure RNA isolation kit (Roche Diagnostics). Samples were quantified using the nanodrop to obtain RNA concentrations. Once the RNA concentrations for 2 × 10^6^ cMSCs was determined, cMSC RNA samples were titrated from 2 × 10^6^ cells in serial dilutions down to the equivalent of two cells. Titrated canine RNA samples were placed into 36 or 500 µg murine RNA to represent a low or high background of murine RNA (Figure 3A). Each of the mixed canine and murine RNA samples were used to create cDNA (SuperScript III cDNA kit, Invitrogen). qPCR was performed as described above using canine RPL13Aand cycle threshold data for each cell titration were interpolated to a standard curve in Prism version 8.00 for Mac (GraphPad Software, La Jolla, CA). qPCR amplification of canine RPL13A (Figure 3B) was similar in either the low concentration of background murine RNA (36 μg; red lines) or the high concentration of background murine RNA (500 μg; blue lines). Therefore, the standard curve to interpolate detection of cMSCs at day 10 was based on the cMSC cell titrations mixed with low concentration of murine RNA (Figure 3C).

### Real-Time Quantitative PCR Detection of cMSCs at Day 10

At Day 10, mice (*n* = 5) from cell therapy groups (cMSCs and cMSCs + BMP-2) were terminated. Dissection instruments and the workspace were treated with RNAse Zap (Invitrogen) prior to harvesting calvaria. Individual calvaria were removed using a rotary tool fitted with a 10 mm diameter diamond-cutting wheel. Calvarial samples were washed in PBS and snap frozen in liquid nitrogen (Figure 2A). RNA was extracted the day of calvarial harvest using a Roche High Pure RNA isolation kit (Roche Diagnostics). Samples were thawed in 800 μL lysis buffer in their respective 50 ml conical tubes and were rocked for 30 min at room temperature after the addition of 400 μL of sterile room temperature PBS. The PBS and lysis buffer mixture was then passed through High Pure RNA isolation kit purification columns following manufacturers protocol and then eluted in 50 μL in elution buffer (Roche Diagnostics).

Individual eluents were then assayed for the number of cMSCs present within each calvarial defect using qPCR for the canine housekeeping gene RPL13A. For each sample, 40 ng of RNA was used to generate cDNA using the SuperScript III cDNA kit (Invitrogen). For quantitative RT-PCR, cDNA samples were amplified in a 20 µL reaction containing SYBR Green PCR master mix (Fast SYBR Green, Applied Biosystems) on a CFX96 Real-Time System (Bio-Rad). Threshold cycles for each specimen were plotted against the previously described standard curve (Figures 3A,B) to quantify the number of cMSC in each defect at Day 10. Values were interpolated in Prism version 8.00 for Mac (GraphPad Software).

### Micro-Computed Tomography Analysis

At 28 days, mice (*n* = 5/group) were terminated and calvaria were excised and placed in 10% neutral buffered formalin (NBF) for fixation. Samples were removed from the 10% NBF, dried with a Kimwipe, and wrapped in Parafilm (VWR International, Radnor, PA) in preparation for µCT. Samples were imaged 360° using a 28 kV beam with image pixel size of 16 μM, flat field correction, and frame averaging with a SkyScan 1,275 µCT (Bruker; Germany). Individual tiff files from each scan were converted to axial bitmap files for further reconstruction analysis using NRecon (Micro Photonics, Allentown, PA). To minimize scanning artifacts, misalignment compensation, ring artifact reduction, beam hardening, and cross-sectional rotation were optimized and kept constant for all samples. Hounsfield units (HU) were set between −934 and 6297 HU for all reconstructions. Control groups were used to optimize the scanning and reconstruction parameters. Mimics 20.0 (Materialise, Plymouth, MI) was used to generate 3-D reconstructions and quantify bone healing. First, cylinders were generated on each calvarial defect reconstruction using a 4 mm diameter cylinder placed around the margin of each defect. Thresholding was set to 70–255 HU to capture mineralized woven bone. Data were reported as both volume (mm^3^) and surface area (mm^2^). Threshold settings were determined based on previous work (Samsonraj et al., 2017).

### Histological Analysis

Upon completion of μCT, calvaria were removed from 10% NBF and decalcified in 1 M dibasic EDTA, pH 8.0 (Sigma). The solution was changed every 2 days until decalcification was confirmed by manual palpation and survey μCT. Samples were dehydrated via increasing alcohol gradations, cleared with Sub-X clearing agent (Surgipath Medical Industries, Richmond, IL) and embedded in paraffin (Thermo Scientific Richard-Allan Scientific, San Diego, CA). Paraffin-embedded samples were cut to 4 µm sections and floated onto Gold Seal Ultra stick slides (Thermo Scientific, San Diego, CA). Prior to staining, sections were heated to 60°C for 20 min in deparaffinized HiPur Xylene (Thermo Scientific Richard-Allan Scientific) and rehydrated. For H&E staining, sections were stained in Gill’s hematoxylin II stain and counterstained with Eosin Y (Poly Scientific R and D, Bay Shore, NY) before clearing and dehydration. Masson’s trichrome staining was performed using a commercially available kit (Thermo Scientific Richard-Allan Scientific) following the manufacturer’s protocol. Slides were cover-slipped with mounting medium (Thermo Scientific Richard-Allan Scientific).

### Immunoflourescence for Collagen Type I, III, VI, and XII

Immunoflourescence was performed to determine the presence of Collagen Type I, III, VI, and XII within calvarial defects. Prior to immunofluorescence, slides were heated to 60°C for 20 min, deparaffinized in HiPur Xylene (Thermo Scientific Richard-Allan Scientific) and rehydrated. Antigen retrieval was performed with 20 µL proteinase K (Agilent) per section followed by a 15-min incubation at 37°C (Dawson et al., 2019). Diluted Vectastain ABC Normal Goat serum was used as the blocking reagent [Vectastain Elite ABC Kit (Rabbit-IgG); Vector Laboratories, Burlingame, CA]. Primary antibodies were diluted in 1% BSA in sterile Tris Buffered Saline (TBS) at a dilution of 1:40 for Collagen I (PA127396, Invitrogen), 1:200 for Collagen III (227341AP, Proteintech Group, Inc., Rosemont, IL), 1:200 for Collagen VI (ab6588, Abcam, Cambridge, MA), and 1:200 for Collagen XII (NBP2-57420, Novus). Antibodies were applied at 20 µL per section and incubated overnight at 4°C. The following day slides were washed with 1X Tri-Buffered Saline with 0.1% Tween (TBST) (Dawson et al., 2019). Bound antibodies were detected by goat anti-rabbit Alexaflour 488 (green) conjugated secondary antibody (Invitrogen) at a 1:500 dilution for 1 h at room temperature. Slides were processed with 4′,6-diamidino-2-phenylindole (DAPI, Invitrogen) for 5 min in order to detect nuclei, rinsed in DI-H2O, dried, and mounted with Prolong Gold Antifade mountant (Invitrogen). Representative images were obtained using an Olympus IX70 Fluorescence microscope (Olympus, Tokyo, Japan) and fluorescent microscopy using SPOT software (version 5.1; Sterling Heights, MO).

### Alkaline Phosphatase Activity and BMP-2 Gene Expression

cMSCs and human bone marrow MSCs (hMSCs; hMSC-7, hMSC-25) were used to compare inherent differences in cMSC and hMSC alkaline phosphatase (ALP) activity. Passage 1 cMSCs and hMSCs were thawed and plated as described above to reach 80% confluent P2’s. P2 cMSCs and hMSCs were washed, trypsinized, and plated at 1 × 10^4^ cells/cm^2^ in 12-well plates (Corning) (*n* = 6 wells/condition). P2 cMSC and hMSCs were also reserved and used for RNA extraction for day 0 time point using High Pure RNA Isolation Kit following manufacturers protocol (Roche). Cells were cultured in 10% CCM, osteogenic differentiation medium (ODM) consisting of α-MEM with 10% FBS, 10 μg/ml β-glycerophosphate (Sigma), 50 mg/ml ascorbate-2-phosphate (Sigma), or ODM + 100 ng/μL of recombinant human bone morphogenic protein-2 [rhBMP-2; ODM + 100 BMP-2; R and D Systems (Krause et al., 2011)]. Media were exchanged twice weekly. At 7 days, wells (*n* = 3/condition) were washed twice with PBS and incubated with 500 μL cold (4°C) ALP activity buffer containing 1 mM magnesium chloride (Sigma), 0.1% Triton-X (Sigma), and 100 mM sodium chloride (Sigma) in PBS (Volk et al., 2005; Krause et al., 2011; Bearden et al., 2017; Gasson et al., 2021). 500 μL cold (4°C) ALP substrate, p-nitrophenylphosphate (PNPP, Thermo Fisher, Waltham, MA), was added to each well to initiate the ALP activity reaction. Absorbance for each well was determined at 405 nm in 1-min intervals for 20 min at 37°C using an automated plate reader (Cytation 5, BioTek, Winooski, VT) and Gen5Bio software. Kinetic curves were generated for each well and the ALP activity of each well was calculated by determining the slope of each kinetic curve using a linear curve fit method. ALP activity was normalized to number of cells per well using DNA quantification as previously described (Krause et al., 2011; Bearden et al., 2017).

The remaining wells were washed twice with PBS prior to RNA extraction using the High Pure RNA Isolation Kit (Roche Life Science, Penzberg, Germany). 300 μL of sterile PBS was pipetted into each well followed by manual dissociation of the monolayer with a pipette tip. Monolayers from replicate wells were combined in 1.7 ml tubes (900 μL/tube) and centrifuged for 3 min at 500 g. Supernatant was removed and pellets were resuspended with 200 μL PBS. 400 μL Lysis Binding Buffer was added following manufacturer’s protocol (Roche High Pure RNA Isolation Kit). All samples were treated with DNase to remove contaminating DNA and quantified using a NanoDropTM 1000 Spectrophotometer (ThermoFisher).

Complementary DNA (cDNA) was synthesized from 300 ng RNA (normalized across all samples for each cell preparation) using the SuperScript III RT Kit (Invitrogen) according to the manufacturer’s protocol. Canine BMP-2 cDNA ORF Clone (SinoBiological cat #DG70088-G, Wayne, PA) was used as a positive PCR control. P1 cMSC and canine bone marrow RNA (Zyagen #DR704, San Diego, CA) were also assessed for BMP-2 expression. cDNA was used to perform reverse transcription PCR (RT-PCR) in a 15 μL reaction. Canine and human BMP-2 forward (F) and reverse (R) primer sequences were generated with primer-blast (Ye et al., 2012) and are as follows: canine F 5′ CGG​TCT​CAT​TAC​GGA​GCT​GG 3′, canine R 5′ CTC​CGG​GTT​GTT​TTC​CCA​CT 3′, human F 5′ ATG​CAA​GCA​GGT​GGG​AAA​GT 3′ and human R 5′ TGG​CCT​TAT​CTG​TGA​CCA​GC 3′. Ethidium bromide agarose gel electrophoresis (110 V and 400 amps for 40 min) was used to identify bands for BMP-2 expression at 324 base pairs for canine BMP-2 and 188 bp for human BMP-2 expression.

Identical primer pairs and cDNA samples were used for qPCR of canine or human BMP-2 expression using a CFX96 Real-Time System (Bio-Rad, Hercules, CA). cMSC expression was normalized to the housekeeping genes RPL13A and RPL32 using cDNA extracted from cells used to set up the ALP Assay at day 0 as a baseline (Peters et al., 2007). hMSCs were normalized to β2M and GAPDH housekeeping genes using either hMSC-7 or hMSC-25 days 0 cDNA as a baseline (Kumar et al., 2009; Ye et al., 2012). Once threshold (CT) levels were normalized they were used to provide relative gene expression using the 2^−ΔΔCt^ method (Livak and Schmittgen 2001).

### Statistics

Descriptive statistics were reported as mean ± standard deviation (SD) for all data. Analytical statistics were performed using Prism version 8.00 for Mac (GraphPad). Data were analyzed using either one-way ANOVA with Dunnett’s multiple comparisons post-test or two-way ANOVA with Tukey’s post-hoc test. Significance was established at *p* < 0.05.

## Results

### Detection of cMSCs in Murine Calvarial Defects at Day 10

RNA samples obtained from the treatment groups that received cells (cMSC and cMSCs + BMP-2) were assessed *via* qPCR for cMSCs at Day 10. RNA samples from the positive control group (BMP-2) were used as an internal control to ensure cMSCs were not detected, as this group did not receive cMSCs as treatment. Ten days after defect creation, of the 2 × 10^6^ cMSCs applied to each defect, a miniscule number of cMSCs ranging from 2–20 cells were detected within each defect (Figure 3C). These results indicate that cMSCs, when delivered in a plasma/thromboplastin carrier in a murine calvarial defect model, do not remain within the defect site at relevant numbers for an extended period of time after implantation.

### Micro-Computed Tomography and Histological Analyses

**FIGURE 4:**
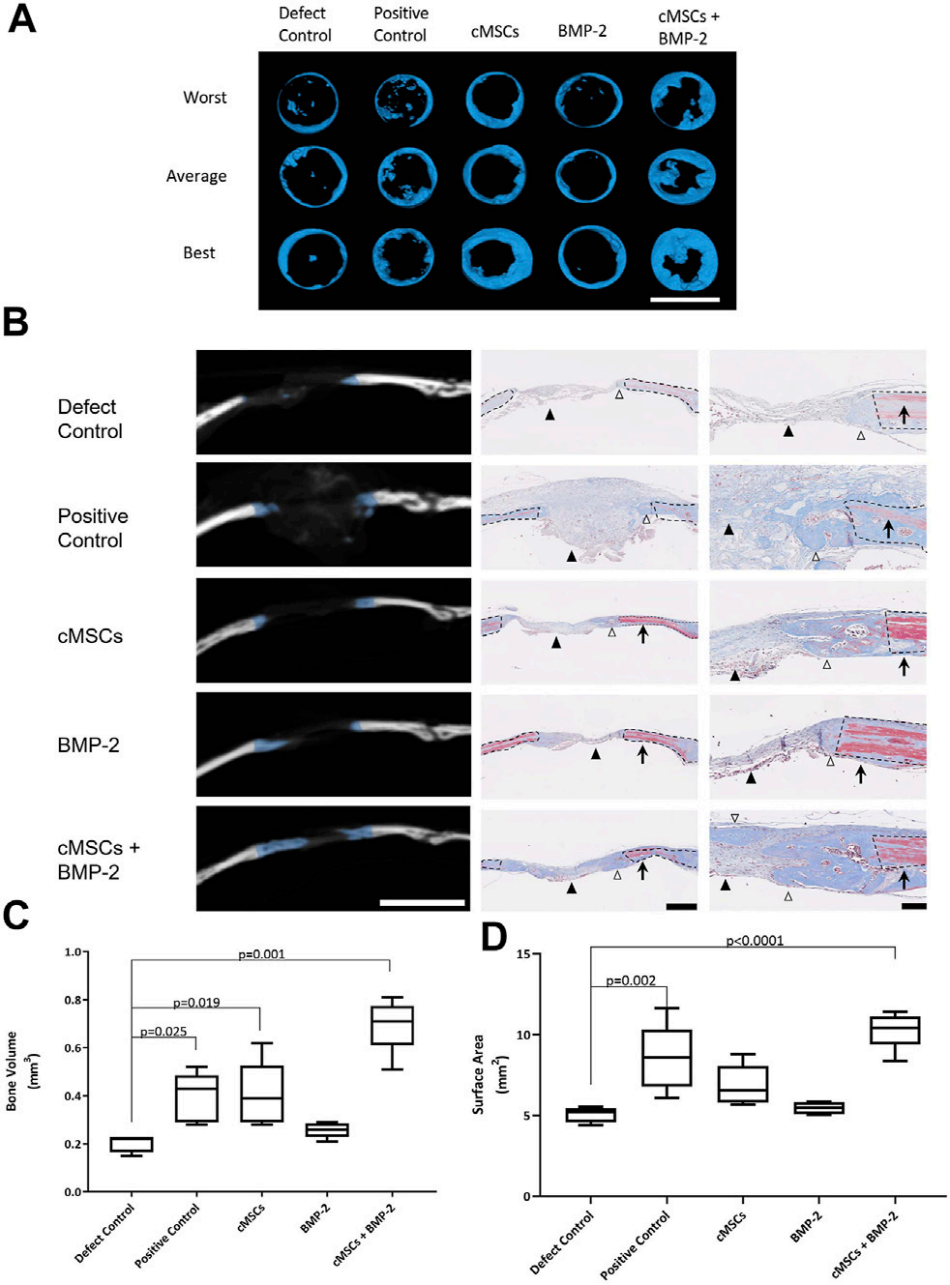
Micro-computed tomography (μCT) and histological analysis of bone healing in murine calvarial defects. **(A)**: μCT was performed at 28 days and used to create coronal plane 3D reconstructions of all calvaria. Reconstructions representing the worst, average, and best bone healing response for each treatment group are provided (bar = 4 mm). Subjectively, the bone healing responses were the greatest for the cMSC and cMSC + BMP-2 treatment groups. **(B)**: Frontal plane μCT images from “average” specimens in panel A are depicted at the widest portion of the defect (bar = 4 mm). Corresponding histology images were obtained at low (bar = 500 μm) or high (bar = 100 μm) magnification. Masson’s Trichrome staining allows identification of fibrous tissue (light blue, black arrowheads), woven bone associated with bone healing (dark blue, white arrowheads), or mature, lamellar bone (red, arrows) adjacent to the calvarial defects. Dashed lines denote the margin of the surgical defect. Consistent with the coronal reconstructions in panel A, the cMSC and cMSC + BMP-2 treatment group exhibited the greatest degree of woven bone formation in the histologic sections, confirming the μCT findings. **(C)**: Quantification of bone volume (mm^3^) at calvarial defect sites are provided for all treatment groups (box and whisker plots denote mean, 25th and 75th percentile, and range). There was no significant difference in bone volume between sub-therapeutic BMP-2 (BMP-2) and the negative control (Defect Control). There was a significant increase in bone volume for the therapeutic concentration of BMP-2 (Positive control), cMSCs alone (cMSCs), and cMSCs with sub-therapeutic BMP-2 (cMSCs + BMP-2). The cMSCs + BMP-2 treatment group exhibited the greatest bone volume of all groups. Data were analyzed with one-way ANOVA followed by Dunnett’s multiple comparison test. **(D)**: Quantification of bone surface area (mm^2^) for all treatment groups as described in panel C. There was no significant difference in bone surface area between cMSCs alone (cMSCs), sub-therapeutic BMP-2 (BMP-2), and negative controls (Defect Control). Bone surface area for the therapeutic concentration of BMP-2 (Positive Control) and cMSCs with sub-therapeutic BMP-2 (cMSCs + BMP-2) was significantly greater than negative controls (Defect Control). The cMSCs + BMP-2 treatment group exhibited the greatest bone surface area of all groups. These results demonstrate that while cMSCs are capable of inducing a bone healing response superior to un-treated defects, co-administration of cMSCs with a sub-therapeutic concentration of BMP-2 initiates a more robust healing response, confirming our hypothesis that cMSCs may require additional agonists to maximize bone regeneration *in vivo*.

All treatment groups were evaluated for bone healing using µCT at Day 28. Coronal plane 3D reconstructions of the best, average, and worst calvarial defects are provided in Figure 4A. Subjectively, there were minimal islands of bone within the center of negative control defects (Defect Control) and the periphery of the defects remained smooth with well-defined margins (Figure 4A). There were increased islands of bone within the defect with some advancement of bone from the periphery of defects in the positive control group (100 μg/ml BMP-2, Figure 4A). Defects treated with cMSCs alone (cMSCs) or cMSCs and sub-therapeutic BMP-2 (cMSCs + BMP-2, Figure 4A) exhibited islands of bone within the defects and circumferential advancement of bone from the defect margins. Defects treated with sub-therapeutic BMP-2 alone (BMP-2) were subjectively similar to the negative controls (Figure 4A). Subjectively, defects treated with cMSCs and the sub-therapeutic concentration of BMP-2 exhibited the greatest degree of bone healing.

In order to assess bone healing histologically, frontal plane histologic sections were generated and assessed in conjunction with matched frontal plane μCT images (Figure 4B). Formation of new, woven bone at the margin of calvarial defects is indicated by blue overlay on the CT images. On adjacent histological sections, defect margins are denoted by black dashed lines. Mason’s Trichrome staining allowed for the differentiation of lamellar bone containing crosslinked, mature collagen at the defect margin (red tissue) or new, woven bone containing more poorly organized collagen and is attempting to heal the defect (dark blue tissue). Histologically, there was fibrous tissue (light blue) with minimal bone formation at the defect margins of negative control defects. In positive controls, the gelatin sponge carrier was readily visible within the defect (light blue), however there was minimal woven bone at defect margins. As with the coronal reconstructions, the most robust bone healing was noted in the groups receiving cMSCs or cMSCs + sub-therapeutic BMP-2. The sub-therapeutic BMP-2 treatment group appeared similar to negative controls.

Bone healing was objectively quantified and results are provided in Figures 4C,D. Bone volume (mm^3^) was as follows: defect control (0.205 ± 0.038), positive control (0.396 ± 0.103), cMSCs (cMSCs; 0.404 ± 0.136), sub-therapeutic BMP-2 (BMP-2; 0.258 ± 0.031), and cMSCs + BMP-2 (0.696 ± 0.111; Figure 4C). Bone surface area (mm^2^) was as follows: defect control (5.06 ± 0.488), positive control (8.57 ± 0.2.06), cMSCs (6.85 ± 1.27), sub-therapeutic BMP-2 (BMP-2; 5.45 ± 0.374), and cMSCs + BMP-2 (10.29 ± 1.15; Figure 4D). Treatment groups that received cMSCs, cMSCs + BMP-2, and the positive controls exhibited significantly higher bone volume when compared to defect controls (*p* = 0.019, *p* = 0.001 and *p* = 0.025, respectively). Positive controls and the treatment group receiving cMSCs + BMP-2 exhibited significantly higher surface area values when compared to untreated controls (*p* = 0.002 and *p* < 0.0001, respectively). Collectively, these results demonstrate that while cMSCs are capable of inducing a bone healing response at Day 28 in an established murine calvarial defect model, the co-administration of cMSCs with a sub-therapeutic concentration of BMP-2 results in a more robust, consistent healing response.

### Immunofluorescence for Collagen Type I, III, VI, and XII

**FIGURE 5:**
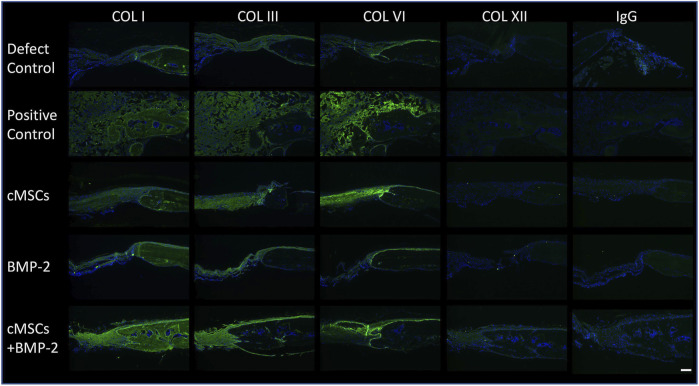
Immunofluorescence of Collagens Type I, III, VI, and XII in murine calvarial defects. Immunofluorescence was performed to determine the presence of Collagen Type I, III, VI, and XII within calvarial defects. Bound primary antibodies were detected with a goat anti-rabbit Alexaflour 488 (green) secondary antibody. Slides were processed with 4′,6-diamidino-2-phenylindole (DAPI) to identify nuclei. Negative control sections (IgG) are provided for reference. Excitation range was 495 nm and emission 519 nm (bar = 100 μM). Representative images are provided for all treatment groups. Collagen Type I, III, and VI were detected in all treatment groups. Collagen I staining was diffusely present, whereas Collagen III was limited to the fibrous tissue bridging the defect and the leading edge of bone healing. Collagen VI was detected in all treatment groups, and co-localized with Collagen III. Collagen XII was not detectible in any of the treatment groups with the exception of the cMSC + BMP-2 treatment group, where it was weakly present and co-localized with Collagens III and VI. Collectively, Collagen I, III, VI, and XII staining was the greatest in the cMSC + BMP-2 treatment group, providing a potential mechanism for the treatment effect of this group.

As exhibited in Figure 3C, we were unable to detect relevant numbers of residual cMSCs within calvarial defects 10 days post-implantation. Despite this finding, treatment of calvarial defects with cMSCs or the combination of cMSCs and sub-therapeutic BMP-2 resulted in the greatest degree of defect healing (Figure 4). For these reasons, we elected to assess calvarial defects for ECM components known to contribute to bone healing (Figure 5). Collagen Type I, III, and VI were detected in all treatment groups. Collagen I was detected within the lamellae of the mature bone adjacent to defects, within the more poorly organized woven bone within healing defects, and sparsely within the fibrous tissue spanning the negative control defects. Subjectively, Collagen I staining was enhanced and diffusely present in the cMSC + BMP-2 treatment group, which is consistent with the enhanced bone healing response in this group. In contrast to Collagen Type I, Collagen III was limited to the fibrous tissue bridging defects or to the leading edge of healing bone, which is consistent with the known role of Collagen Type III in wound and bone healing (Volk et al., 2011; Izu et al., 2012; Garnero 2015; Miedel et al., 2015). Collagen III was subjectively increased in the cMSC + BMP-2 treatment group. Collagen VI exhibited a similar immunofluorescence pattern to Collagen III. Collagen VI was present in all treatment groups and was localized to the fibrous tissue bridging the center of calvarial defects or to the leading edge of healing bone, with the greatest degree of staining in the positive control, cMSC, and cMSC + BMP-2 treatment group. Collagen XII was not detectible within any of the treatment groups with the exception of the cMSCs + BMP-2 group in which it was weakly present both within the fibrous tissue bridging the defects and the leading edge of healing bone. In summary, Collagen I, III, VI, and XII exhibited distinct deposition patterns (Collagen I vs. III) and exhibited the greatest degree of staining in the cMSC + BMP-2 treatment group. These results suggest that enhanced ECM deposition of anabolic collagens may be one method by which cMSCs, which are transiently present within the defects, may enhance bone healing *in vivo*.

### ALP Activity and BMP-2 Gene Expression

**FIGURE 6:**
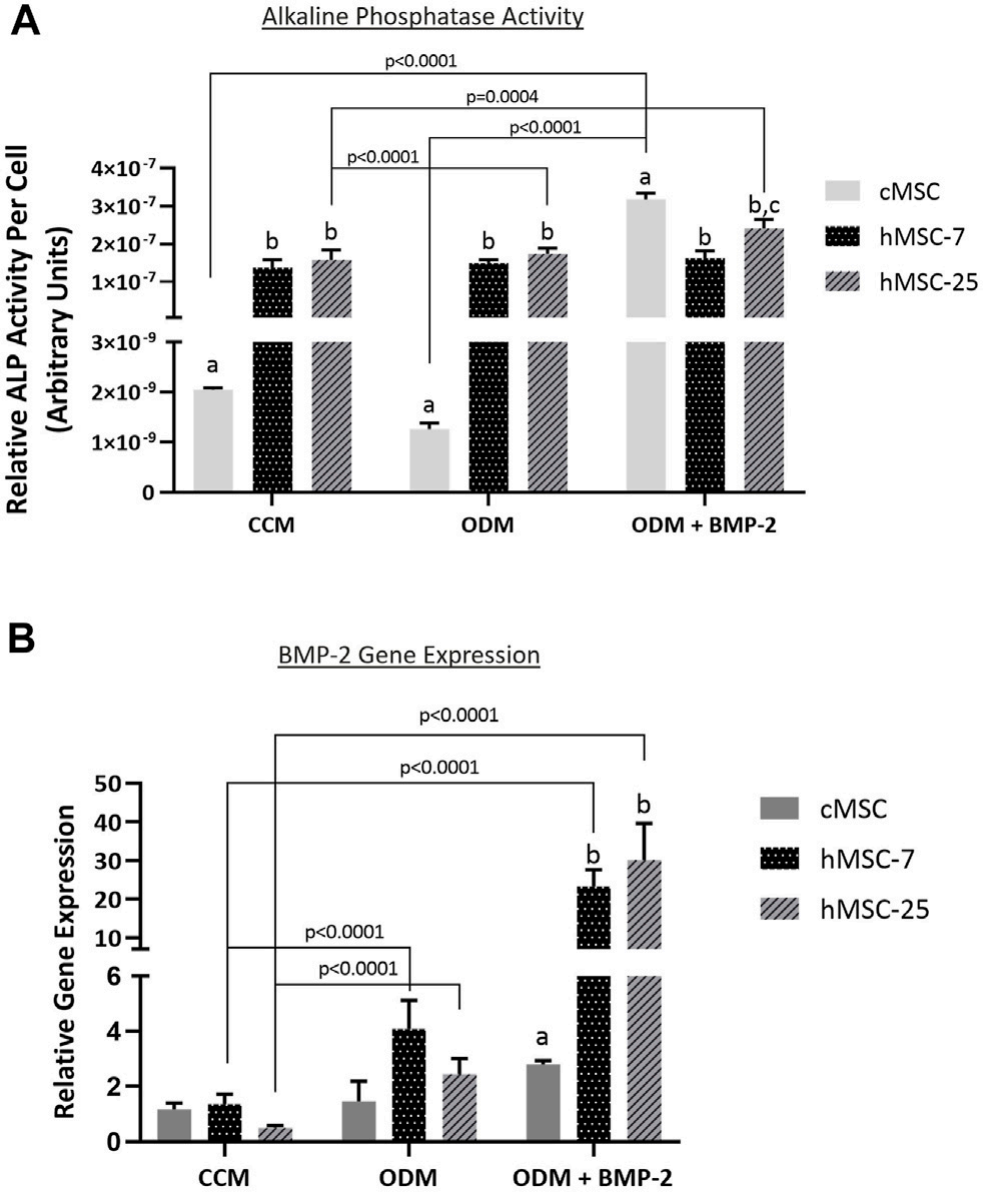
Alkaline phosphatase (ALP) activity and endogenous BMP-gene expression in cMSCs and hMSCs. **(A)**: cMSCs and two preparations of hMSCs were examined using the classic ALP activity assay. Cells were cultured for 7 days in control (CCM), osteogenic medium (ODM), or osteogenic medium containing 100 ng/ml BMP-2 (ODM + BMP-2). ALP activity for each well was normalized to cell number by DNA quantification. Data were analyzed with two-way ANOVA and Tukey’s post hoc test. cMSCs exhibited minimal ALP activity in CCM or ODM, but responded robustly when cultured in ODM + BMP-2. In contrast, ALP activity for both preparations of hMSCs was robust regardless of culture conditions. These results are consistent with prior work and demonstrate important differences between cMSC and hMSC *in vitro* osteogenic differentiation. **(B)**: qPCR results for canine and human bone morphogenic protein-2 (BMP-2) from the ALP activity assay in Panel A. Data were analyzed with two-way ANOVA and Tukey’s post hoc test. In support of the ALP activity assay results, cMSCs did not initiate a significant increase in BMP-2 gene expression in response to ODM or ODM + BMP-2. In contrast, both preparations of hMSCs demonstrated significantly greater BMP-2 gene expression when cultured in ODM or ODM + BMP-2. Lastly, there was significantly reduced canine BMP-2 gene expression as compared to the human BMP-2 gene expression when cells were cultured in ODM + BMP-2.

In order to further characterize these *in vivo* findings, ALP activity and endogenous BMP-2 gene expression were assessed in both cMSCs and hMSCs. Consistent with prior studies, cMSCs exhibited minimal ALP activity when cultured in control (CCM) or basal osteogenic medium (ODM). There was a significant increase in cMSC ALP activity in the presence of osteogenic medium containing BMP-2 (ODM + BMP-2) (Figure 6; *p* < 0.0001). (Bearden et al., 2017). In contrast, two preparations of hMSCs (hMSC-7 and hMSC-25) exhibited robust ALP activity in all media conditions, similar to previously published work (Diefenderfer et al., 2003a; Diefenderfer et al., 2003b; Clough et al., 2015) These results suggest that hMSC basal ALP activity is constitutively active, whereas cMSC ALP activity requires osteogenic induction media containing BMP-2.

To determine whether endogenous BMP-2 gene expression may play a role in this finding, Day 7 RNA samples were extracted from triplicate wells from the ALP assay in Figure 6A and assessed for canine or human BMP-2 expression using qPCR (Figure 6B). Gene expression of canine BMP-2 was minimally affected by osteogenic induction media. Expression of human BMP-2 was significantly increased when hMSCs were cultured in osteogenic induction medium (ODM) or medium containing BMP-2 (ODM + BMP-2). The relative expression of human BMP-2 was significantly greater than canine BMP-2 in the OBM + BMP-2 condition. These qPCR results indicate that the preparation of cMSCs used in the present study do not significantly increase endogenous canine BMP-2 expression in response to osteogenic induction media, even in the presence of BMP-2.

**FIGURE 7:**
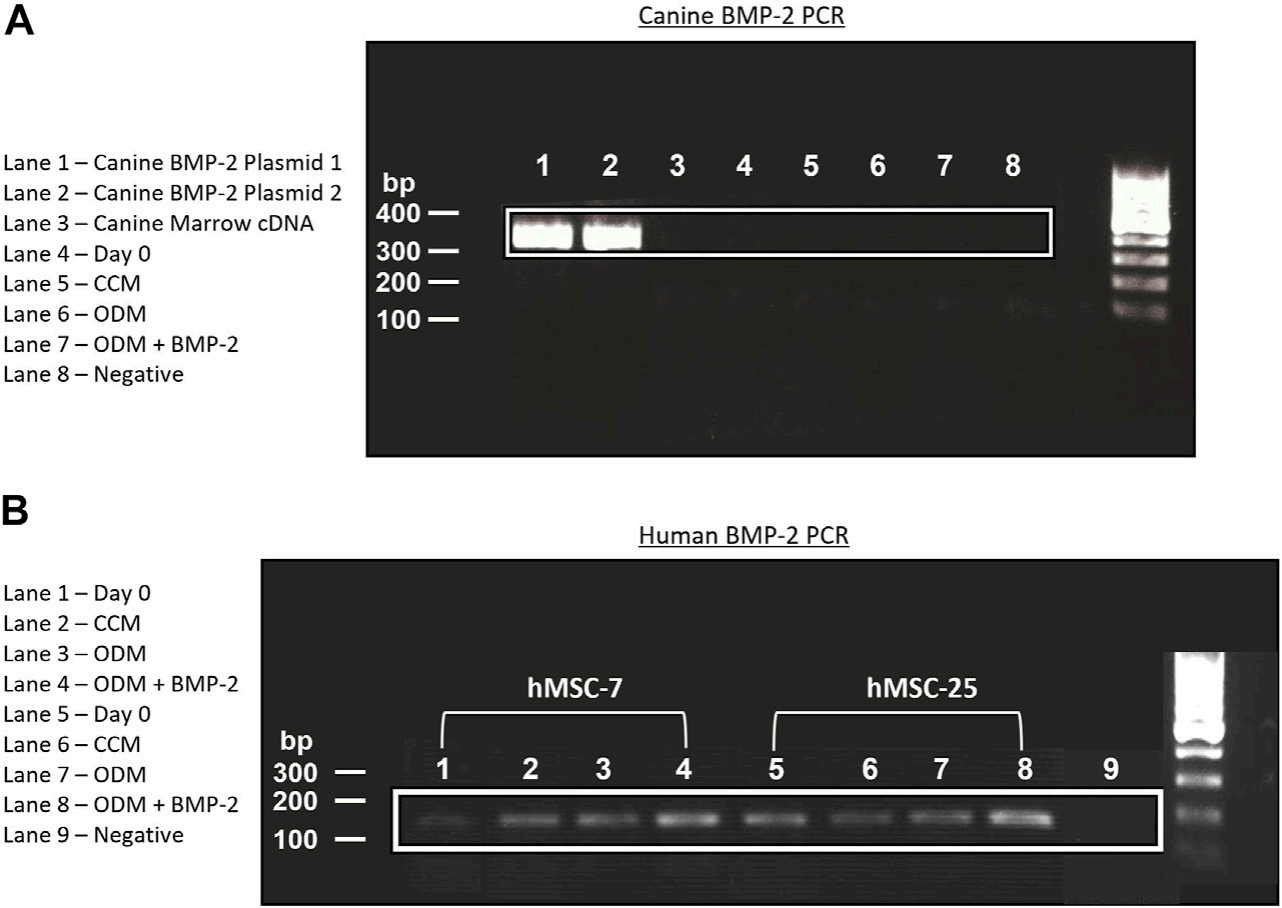
Endogenous expression of canine and human BMP-2 by RT-PCR. **(A)**: A plasmid encoding the canine BMP-2 gene was acquired and two preparations of this plasmid cDNA were used for positive controls (Lanes 1, 2). RNA from canine bone marrow, or RNA samples obtained from cMSCs at Day 0 or Day 7 of the ALP activity assay (Figure 6) were used to create cDNA. All cDNA samples were assessed for canine BMP-2 gene expression using a primer pair designed to create a 324 bp transcript. There was robust expression of the canine BMP-2 gene in the control plasmids (Lanes 1 and 2). Canine bone marrow and cMSCs at Day 0 and Day 7 of the ALP activity assay did not exhibit detectible canine BMP-2 gene expression. **(B)**: RNA from the two preparations of hMSCs in Figure 6 were used to create cDNA. Expression of human BMP-2 was evaluated in both preparations of hMSCs at Day 0 and Day 7 (CCM, ODM, and ODM + BMP-2) with a primer pair designed to create a 188 bp transcript. Human BMP-2 was present at both timepoints and in all conditions. There was an increase in BMP-2 signal in the samples isolated from Day 7 of the ALP activity assay, particularly in the cells treated with ODM + BMP-2. These findings are supportive of the results provided in Figure 6 and demonstrate clear differences in endogenous BMP-2 gene expression of canine and human MSCs in response to osteogenic induction media.

One explanation for the lack of endogenous BMP-2 expression in the cMSCs is that the cMSCs initially possessed the ability to express endogenous BMP-2, but lost this ability during isolation, culture expansion, or cryopreservation (Hass et al., 2011; Yang et al., 2018). Adult, canine, bone marrow cDNA and cDNA samples from P2 cMSCs at Day 0 or Day 7 of the ALP activity assay were assessed for BMP-2 expression using RT-PCR. A plasmid containing canine BMP-2 was used as a positive control (Figure 7A). RT-PCR of the positive control plasmid produced the expected canine BMP-2 band at 324 base pairs. There was no visible expression of canine BMP-2 in canine bone marrow, P2 cMSCs at Day 0 of the ALP activity assay, or P2 cMSCs after 7 days of culture in CCM, ODM, or ODM + BMP-2. These results suggest that canine BMP-2 is not expressed in bone marrow isolated from adult canines, nor is BMP-2 expressed in isolated, low passage cMSCs cultured in control or osteogenic induction media (with or without BMP-2).

Human BMP-2 gene expression was also evaluated using RT-PCR (Figure 7B) for the two preparations of hMSCs used for comparative purposes in the ALP activity assay (Figure 6). In contrast to the cMSCs in which we were unable to detect canine BMP-2 expression, hMSCs expressed BMP-2 on Day 0 of the ALP activity assay, as well as after 7 days of culture in CCM, ODM, or ODM = BMP-2). Subjectively, there was increased expression in the hMSCs after 7 days of culture in ODM (Figure 7B; Lanes 3,7) and ODM + BMP-2 (Figure 7B; Lanes 4,8), consistent with the qPCR results reported in Figure 6B. RNA samples extracted from hMSCs at Day 0 (when cells were plated for ALP assay) exhibited slightly reduced BMP-2 expression. Negative controls did not produce BMP-2 bands (Figure 7B; Lanes 1,5, and 9). These RT-PCR results suggest that in contrast to cMSCs, hMSCs are capable of endogenous BMP-2 expression, and furthermore, that BMP-2 expression is increased when hMSCs are cultured in the presence of osteogenic induction media.

## Discussion

The dog represents a strong translational model for cell-based bone-healing (Hoffman and Dow 2016). However, key similarities and differences between canine and human MSC osteogenic differentiation must be defined if this model species is to be used most effectively. The primary objective of the present study was to determine if previous *in vitro* findings regarding cMSC osteogenic differentiation were relevant in the *in vivo* setting, specifically the observations by multiple investigators that cMSCs do not undergo robust osteogenic differentiation unless supplemented with additional growth factors such as IGF-1, NELL-1, or BMP-2 (Volk et al., 2005; Levi et al., 2011; Bearden et al., 2017; James et al., 2017; Gasson et al., 2021). A secondary objective was to characterize osteogenic differentiation and endogenous BMP-2 gene expression *in vitro* for both cMSCs and hMSCs. Using an established murine calvarial defect model, defects treated with cMSCs in a murine plasma carrier exhibited a modest bone healing response. As expected, defects treated with a sub-therapeutic concentration of BMP-2 (6 μg/ml) were not significantly different than negative controls. However, when cMSCs were co-administered with BMP-2, there was a marked increase in defect healing when assessed by quantitative μCT and histology. Interestingly, relevant numbers of cMSCs were not detectible in calvarial defects 10 days after implantation, suggesting that the cMSC and BMP-2 healing response was driven by paracrine cues or ECM-mediated signals. To that end, Collagen I, III, VI, and XII immunofluorescence was the greatest in defects treated with cMSC and BMP-2, providing evidence that key anabolic ECM-mediated signals were involved in the improved healing response. Consistent with prior work (Volk et al., 2005; Levi et al., 2011; Bearden et al., 2017; James et al., 2017; Gasson et al., 2021), cMSCs failed to undergo early-stage *in vitro* osteogenic differentiation when cultured under typical osteo-induction conditions and required supplementation of osteogenic medium with BMP-2 in order to exhibit ALP activity. Lastly, cMSCs did not express canine BMP-2 in response to osteogenic induction under any treatment condition, whereas hMSCs expressed human BMP-2 under control and osteogenic differentiation conditions.

When compared to the human MSC field, much less attention has been given to cMSCs. While some studies suggest that cMSCs undergo *in vitro* osteogenic differentiation in a manner similar to hMSCs, a growing body of work indicates cMSCs require additional agonists to consistently initiate osteogenic differentiation. Volk and colleagues initially reported that cMSCs isolated from bone marrow of multiple dogs exhibited little ALP activity unless the osteogenic medium was supplemented with BMP-2 and ascorbate-2-phosphate (Volk et al., 2005). This finding was subsequently confirmed in a large scale characterization study of cMSCs derived from synovial, adipose, or bone-marrow tissue (Bearden et al., 2017). While BMP-2 has most often been used to supplement cMSC osteogenic differentiation, other agonists such as NELL-1 and IGF-1 have also been used to improve *in vitro* osteogenic differentiation of cMSCs (Levi et al., 2011; James et al., 2017).

In a study evaluating the *in vitro* and *in vivo* osteogenic potential of adipose derived MSCs from murine, canine, and human donors, Levi and colleagues reported that cMSCs exhibited poor osteogenic differentiation *in vitro* unless osteogenic differentiation medium was supplemented with IGF-1 (Levi et al., 2011). Furthermore, cMSCs failed to induce healing of critically-sized murine calvarial defects whereas hMSCs induced a strong healing response. Based on the study results, it was suggested that cMSCs may need to be primed with IGF-1 or other agonists prior to *in vivo* administration, drawing into question the relevance of the canine translational model in bone healing studies.

There are important differences in the effective dose of recombinant human (rh)BMP-2 across species (Faria et al., 2007; Schmiedt et al., 2007). In rodents, it was previously shown that BMP-2 at a concentration of 100–160 μg/ml was optimal for inducing spinal fusion (Bae et al., 2013; McNeill et al., 2020). In a stepwise dilution experiment, it was determined that administration of 6 μg/ml BMP-2 was sub-therapeutic and did not lead to formation of *de novo* bone or spinal fusion. However, when 6 μg/ml of BMP-2 was co-administered with allogenic bone marrow aspirate into fusion sites, 89% of the sites achieved stable fusion. The results of Levi et al. as well as Bae et al., when considered in the context of prior work from our lab (Bearden et al., 2017; Gasson et al., 2021) collectively inspired the present study. The concentrations of BMP-2 selected in the present study for positive control (100 μg/ml) and sub-therapeutic (6 μg/ml) BMP-2 treatment groups were selected based on the results of Bae et al. Consistent with their findings, murine calvarial defects treated with 6 μg/ml BMP-2 alone did not have significantly different bone healing as compared to negative controls, supporting the use of this concentration of BMP-2 as a sub-therapeutic dose in the murine calvarial defect setting.

In order to provide some insight into the mechanism by which cMSCs contribute to bone healing in this model, mice were terminated 10 days post treatment and calvaria were examined for residual cMSCs using species-specific qPCR (Figure 2A). Murine and canine primers were developed to the housekeeping genes RPL13A and GAPDH (Peters et al., 2007; Ye et al., 2012). Primers were validated and dilutional experiments were performed in a background of low or high concentrations of murine RNA to detect RNA from 2 × 10^6^ down to as few as two cMSCs (Figures 2, 3). Using this technique, we were unable to detect relevant numbers of cMSCs within calvarial defects 10 days after implantation (Figure 3C), despite the fact that bone healing was observed in response to these two treatment groups (cMSCs alone, cMSCs with sub-therapeutic BMP-2).

It was initially believed that MSCs homed to site of injury, differentiated into cells of need, and permanently engrafted to survive as reparative tissue (Prockop et al., 2003; Caplan 2005). A more recent proposed mechanism of action is that of the MSC as a medicinal supply cell or nurse cell. Under this concept, MSCs respond to local injury cues and produce autocrine and paracrine signals such as cytokines, growth factors, and ECM components that improve the host’s endogenous healing abilities (Osugi et al., 2012; Freitas et al., 2019). Putative factors in the medicinal supply cell paradigm, include, but are not limited to, vascular endothelial growth factor (VEGF), basic fibroblast growth factor (bFGF), insulin-like growth factors (IGFs), hepatocyte growth factor (HCF), platelet-derived growth factor-ββ (PDGF-ββ), BMP-2, and stromal cell-derived factor-1 (SDF-1), and various provisional ECM components. Results of the present study suggest that cMSCs, when administered in a murine plasma carrier, likely initiate bone healing through this mechanism.

While much effort has been focused on administration of MSCs to accomplish skeletal regeneration, recent focus in the field has shifted away from cell delivery to the administration of MSC-derived exosomes or ECM components (Osugi et al., 2012; Zeitouni et al., 2012; Clough et al., 2015; McNeill et al., 2020). One compelling strategy to enhance bone healing in the clinical setting is the delivery of anabolic MSC-derived ECM scaffolds at the time of surgical stabilization. In support of this concept, it has been previously shown that MSCs can be induced to deposit anabolic ECM on gelatin surgical sponges and that these deposits contain a number of important collagens known to stimulate bone formation, namely Collagens I, III, VI, and XII (Zeitouni et al., 2012). This approach also resulted in improved healing of critically sized murine femoral defects (Clough et al., 2015) and when co-administered with hMSCs led to the greatest degree of defect healing. These studies provide evidence that anabolic ECM derived from osteogenically differentiated MSCs are capable of initiating bone healing even in the absence of cells. The authors of these studies hypothesized that Collagen VI and XII may serve as key biomarkers for treatment success. Collagen VI and XII have also been shown to co-localize to inter-cellular contacts and interference with Collagen VI or XII expression resulted in altered deposition of these collagens both within and adjacent to MSCs (Izu et al., 2016; McNeill et al., 2020).

Results of the present study provide additional evidence that Collagens I, III, VI, and XII are key ECM components for robust bone regeneration. While Collagen VI was detected in most treatment groups, the greatest degree of deposition was noted in the cMSC + BMP-2 treatment group, which was the only group in which Collagen XII was detectible. This treatment group also exhibited the greatest degree of bone healing. Collagen VI and XII have been shown to regulate development of osteoblasts during murine skeletal morphogenesis (Izu et al., 2012). Thus, the observation that Collagen VI and XII immunofluorescence were enhanced in the treatment group with the greatest degree of bone healing is consistent with what is currently known about these collagens. One potential mechanism to explain the effect of cMSCs and sub-therapeutic BMP-2 in the present study is the binding affinity of Collagen VI with BMP-2. cMSCs administered to the defects may have initiated increased Collagen VI deposition, which led to binding of BMP-2, trapping of BMP-2 in provisional ECM, and potentiating BMP-2 effects (McNeill et al., 2020).

The findings in the present study that cMSCs fail to initiate ALP activity in response to osteogenic induction media unless supplemented with BMP-2 is consistent with prior work (Volk et al., 2005; Bearden et al., 2017; Gasson et al., 2021). To the authors’ knowledge, however; marrow-derived cMSCs have not been evaluated for expression of endogenous (canine) BMP-2 in response to osteogenic stimuli. While two different preparations of hMSCs expressed endogenous BMP-2 and expression was increased in response to osteogenic media, we were unable to detect endogenous expression of canine BMP-2 in cMSCs under any setting with the exception of a control plasmid. Moreover, we assessed canine bone marrow cDNA for BMP-2 gene expression and none was detected. The canonical Wnt/β-catenin pathway and the BMP/SMAD signaling pathways are both known to regulate osteoblastic differentiation (Yang et al., 2015; Zhao et al., 2020; Zhang et al., 2013). Complex, incompletely characterized cross-talk occurs between these two pathways. It is presently unclear whether the BMP-2 pathway is capable of providing upstream feedback to Wnt/β-catenin (Yang et al., 2015; Zhao et al., 2020), or whether Wnt/β-catenin is upstream of BMP-2 signaling (Zhang et al., 2013). There are likely multiple levels of cross-talk between the two pathways. The results presented in this study may suggest that in cMSCs, BMP-2 signaling functions to activate an insufficient or partially inhibited Wnt/β-catenin pathway. Another potential explanation is that cMSCs may express increased levels of Dkk-1, which is a known inhibitor of the Wnt/β-catenin pathway. Interestingly, Dkk-1 is unable to inhibit the Wnt pathway when it is transactivated by exogenous BMP-2 administration (Zhang et al., 2013).

It is presently unclear why the cMSCs examined in this study are incapable of expressing endogenous canine BMP-2. The RT-PCR results in which canine adult bone marrow cDNA were examined for canine BMP-2 expression suggest that the cMSCs did not lose the ability to express BMP-2 after isolation or cell expansion in response to an altered *in vitro* niche. As mentioned above, dogs respond to recombinant BMP-2 therapy in both induced- and spontaneous-injury settings (Faria et al., 2007; Boudrieau 2015). Due to the non-specific nature of BMP-2 receptor binding, it is possible that canine-derived cMSCs express a complete set of Type-I and -II BMP receptors but that in the canine species, other endogenous BMP’s that are associated with skeletal development such as BMP-4, -6, or -7 are more physiologically relevant (Kim et al., 2018; Teunissen et al., 2018). The signaling cross-talk between canine BMP/SMAD and Wnt/β-catenin and their respective roles in cMSC osteogenic differentiation as well as endogenous BMP expression will be the focus of future studies.

As with all studies, the present study is not without limitations. While providing important *in vivo* support for previous *in vitro* findings, a single preparation of canine bone marrow-derived MSCs was used. This preparation of cMSCs was selected due to the fact that these cMSCs exhibited average *in vitro* osteogenic differentiation parameters and as such were representative of canine marrow MSCs. RNA extractions of harvested murine calvaria were used to screen for residual cMSCs 10 days post-implantation. It is possible that some cMSCs were present within deeper portions of healing osteoid and were not recovered during RNA extractions, although the extraction protocol described above resulted in visible removal of all soft and some hard tissues from the defect sites. The immunofluorescence performed in the present study, while providing support for the concept of cMSC-driven ECM deposition of anabolic collagen molecules, did not exhaustively characterize all ECM components known to be stimulatory or inhibitory to the bone healing response. Documentation that cMSCs are unable to express endogenous BMP-2 represents an important observation, but the mechanistic explanations for this observation remain to be determined and will be the focus of future work. Lastly, while the present work represents an important *in vivo* finding, the athymic calvarial defect model is not representative of the more complex body systems of larger animals such as dogs or humans that contain well-developed immune systems and substantially different biomechanical environments.

In conclusion, this study demonstrates important differences between cMSC and hMSC *in vitro* osteogenic differentiation and endogenous BMP-2 expression. More importantly, it provides the first *in vivo* evidence that cMSCs may require supplemental agonists such as BMP-2 to accomplish *in vivo* healing. This strategy may also prove useful in order to reduce the cost and side effects associated with administration of high concentrations of BMP-2 (Carragee et al., 2011; Faundez et al., 2016).

## Data Availability

The raw data supporting the conclusion of this article will be made available by the authors, without undue reservation.
